# Rapamycin protects against gentamicin-induced acute kidney injury via autophagy in mini-pig models

**DOI:** 10.1038/srep11256

**Published:** 2015-06-08

**Authors:** Jing Cui, Xue-Yuan Bai, Xuefeng Sun, Guangyan Cai, Quan Hong, Rui Ding, Xiangmei Chen

**Affiliations:** 1 Department of Nephrology, Chinese PLA General Hospital, Chinese PLA Institute of Nephrology, State Key Laboratory of Kidney Diseases, National Clinical Research Center for Kidney Diseases, Beijing, China

## Abstract

Gentamicin may cause acute kidney injury. The pathogenesis of gentamicin nephrotoxicity is unclear. Autophagy is a highly conserved physiological process involved in removing damaged or aged biological macromolecules and organelles from the cytoplasm. The role of autophagy in the pathogenesis of gentamicin nephrotoxicity is unclear. The miniature pigs are more similar to humans than are those of rodents, and thus they are more suitable as human disease models. Here we established the first gentamicin nephrotoxicity model in miniature pigs, investigated the role of autophagy in gentamicin-induced acute kidney injury, and determined the prevention potential of rapamycin against gentamicin-induced oxidative stress and renal dysfunction. At 0, 1, 3, 5, 7 and 10 days after gentamicin administration, changes in autophagy, oxidative damage, apoptosis and inflammation were assessed in the model group. Compared to the 0-day group, gentamicin administration caused marked nephrotoxicity in the 10-day group. In the kidneys of the 10-day group, the level of autophagy decreased, and oxidative damage and apoptosis were aggravated. After rapamycin intervention, autophagy activity was activated, renal damage in proximal tubules was markedly alleviated, and interstitium infiltration of inflammatory cells was decreased. These results suggest that rapamycin may ameliorate gentamicin-induced nephrotoxicity by enhancing autophagy.

Acute kidney injury (AKI) is a major kidney disease characterized by rapid loss of renal function, resulting in accumulation of metabolic waste and imbalanced electrolytes and bodily fluid^1^. AKI is increasingly prevalent in both developing and developed countries, and is associated with severe morbidity and mortality^2^. The incidence of AKI is reported to be >14%, with hypovolemia, nephrotoxic drugs, cardiac dysfunction and respiratory failure being the most common etiologies^3^,^4^. Drug-induced kidney injury is becoming a major cause of AKI in China^5^.

Aminoglycosides are antibiotics used in clinical settings to treat serious infections due to Gram-negative bacteria. Nephrotoxicity due to aminoglycosides occurs in 10–20% of therapeutic regimes. An important part of gentamicin-induced AKI is related to its tubular effect, which is triggered by drug accumulation in epithelial tubular cells. The pathogenesis of gentamicin nephrotoxicity is not clear. Studies showed that gentamicin acts on mitochondria and induces oxidative stress and apoptosis. Ultimately, gentamicin causes glomerular congestion, renal free radical generation, reduced antioxidant defense mechanisms, and acute tubular necrosis^6^,^7^,^8^,^9^.

Autophagy is a highly conserved physiological process which might remove damaged or aged biological macromolecules and organelles from the cytoplasm^10^. Although autophagy regulates many critical aspects of normal and disease conditions in the kidneys, such as tubular injuries, kidney development and aging^1^,^11^,^12^, the importance of autophagy in the kidneys has only just begun to be elucidated. How the process of autophagy is altered in the pathogenesis of renal diseases and how this alteration is beneficial or detrimental to renal functions is unclear^13^. The mammalian target of rapamycin complex 1 (mTORC1) inhibitor, rapamycin, stimulates autophagy. In a mouse model of cisplatin nephrotoxicity, pharmacologic blockade of autophagic flux by chloroquine significantly enhanced cisplatin-induced kidney injury, whereas activation of autophagy by rapamycin protected proximal tubules from injury^1^. Currently, however, the role of autophagy in gentamicin-induced AKI and the effect of rapamycin on renal injury are unclear.

Generally, animal testing in drug development studies requires at least two species, most typically rats and dogs; non-human primates and guinea pigs are also commonly used^14^. Compared with rodents, the miniature pig (mini-pig) is more similar to humans in terms of anatomy, physiology and genetics. For example, trinucleotide repeat regions are more highly conserved between humans and pigs than between humans and rodents^15^. Results obtained from pharmaceutical experiments in mice have led to various types of damage to humans, while the results obtained from experiments in miniature pigs resemble those in humans. For example, streptomycin, another aminoglycoside antibiotic, is a commonly used drug for the treatment of tuberculosis and other infectious diseases, but it causes damage to the patient’s vestibular and auditory cells. In mouse models, streptomycin does not have these side effects, but in miniature pigs, streptomycin has toxic side effects similar to those observed in humans^16^. Therefore, in recent years, large animals, such as the miniature pig, have been used to establish a variety of human disease models^17^,^18^. The use of miniature pigs also has advantages in terms of ethics and costs.

In this study, we used the Chinese Experimental Miniature Pig from the China Agricultural University (Beijing) to establish a gentamicin-induced AKI model. The Chinese Experimental Miniature Pig was derived from little swine of the Guizhou Province, China, in 1985, and its genetic background is well understood. Its characteristics include an inherently small size, early sexual maturity, rapid breeding and ease of management. In addition, it maintains the genetic integrity and uniformity of the population. Further, we investigated the role of autophagy, particularly mitochondrial autophagy and oxidative stress, in the pathogenesis of gentamicin-induced nephrotoxicity. Finally, we investigated the effect of rapamycin on renal function, autophagy, apoptosis and inflammation in the mini-pig gentamicin-induced AKI.

## Materials and Methods

### Animals and environmental conditions

The male Chinese Experimental Mini-pigs aged 12 weeks were obtained from the State Key Laboratory for Agrobiotechnology in China Agricultural University. Two animals per cage were housed in a room maintained at a temperature of 18–22 °C and a relative humidity of 30–70% with artificial lighting from 8:00 to 20:00 hours. The experimental protocol was carried out in accordance with the approved guidelines of the Institutional Animal Care and Use Committee at the Chinese PLA General Hospital.

### Experimental groups and treatment

Gentamicin (GM) was purchased from Guangzhou Baiyunshan Tianxin Phamarceutical Co. LTD and rapamycin was purchased from East China Pharmaceutical Limited CO. LTD. In the dose-response study of gentamicin, minipigs received a daily i.m. injection for 10 days of gentamicin sulfate (5, 6.85, 10, 15, 20, 40, 60, 80, 100 and 120 mg/kg) in distilled water. A total of 33 healthy male minipigs were randomly assigned to 11 experimental groups (n = 3 per group). In the 120 mg/kg group, the miniature pigs died 1 day after injection. The 6.85 mg/kg is the clinically relevant doses of gentamicin. For the gentamicin time course study, minipigs received an i.m. injection of 80 mg/kg gentamicin daily for 0, 1, 3, 5, 7 and 10 days. To test the effect of rapamycin in gentamicin-induced acute kidney injury, first group is the control animals, second is the one that was treated with GM (80 mg/kg/i.m./10 days), third is the one that was treated with GM (80 mg/kg/i.m./10 days) and rapamycin (0.3 mg/kg/p.o./10 days).

### Serum biochemistry analysis

At the scheduled termination day, all minipigs were euthanized by pentobarbital, and serum samples were collected by centrifugation at 3,000 rpm for 10 min and stored in the −80 °C freezer before analyzing. Serum biochemical parameters were analyzed by an autoanalyzer (Cobas8000, Roche, Germany).

### Histological and TUNEL assay

The kidneys from pigs were excised, fixed in 4% paraformaldehyde, embedded in paraffin and sectioned at 4 μm for histological staining with periodic acid-Schiff. Kidney sections were mounted on glass slides and stained with hematoxylin and eosin for histopathologic evaluation. Acute tubular necrosis (ATN) scores were assigned semiquantitatively by light microscopy on a scale of 0–4: 0 = normal histology; 1 − 4 = <25, <50, <75, and >5%, respectively, of epithelial cells of the proximal convoluted tubules showing necrosis, degeneration, regeneration, tubular dilatation, protein casts, and interstitial lymphocytic infiltration^19^. Apoptosis in renal tissues was identified by a TUNEL assay with *In Situ* Cell Death Detection Kit (Roche, 11684817910). Some tissue sections were subjected to antigen retrieval by microwaving for 10 min in 10 mM sodium citrate buffer [pH 6.0]. Endogenous peroxidase activity was blocked by incubation with 3% hydrogen peroxide for 30 min. After PBS washing, sections were incubated with 1.5% normal goat serum for 30 min, followed by incubation with TUNEL reaction mixture overnight at 4 °C. After three washes with PBS, the samples were incubated with converter-POD at room temperature. After PBS washing, the sections were incubated with DAB followed by examination under the microscope. For estimation of the level of apoptosis, the TUNEL positive renal tubular epithelial cells, as well as unstained cells, were scored in 10 different fields at 400 magnification. The apoptotic index was expressed as the percentage of total cells scored. The results were expressed as the average number of TUNEL-positive cells for each group. All counting procedures were performed blindly.

### RNA isolation and real-time quantitative PCR

Total RNA was isolated from renal tissues using TRIzol (Invitrogen, Carlsbad, CA, USA) following the manufacturer’s instructions. Reverse transcription was performed using a TIANScript RT kit (Tiangen Biotech, KR104). Amplification was performed in a 7500 real-time PCR System (Applied Biosystems). Reaction contained 50 ng total cDNA, 0.2 μM primers, and 10 μL 2 × SYBR green buffer (TaKaRa, DRR820A) in a final volume of 20 μL. Primers were designed using the software package Primer Express 2.0 (Applied Biosystems) based on GenBank nucleotide sequences as follows:

CCL-5(Accession: NM_001129946.1): Forward 5’-GTGTGTGCCAACCCAGAGAA -3’, Reverse 5’-GGACAAGAGCAAGAAGCAGTAGG -3’. ICAM-1(Accession: NM_213816.1): Forward 5’-ACCCACC CACACCTTGCTAC -3’, Reverse 5’-GCTGGGAACAGTCCATCCA-3’. IL-6(Accession:JQ839263.1): Forward 5’-GGGAAATGTCGAGGCTGTG-3’, Reverse 5’-AGGGGTGGTGGCTTTGTCT-3’. MCP-1(Accession:NM_213816.1): Forward 5’-GGGTATTTAGGGCAAGTTAGAAGGA-3’, Reverse 5’-CATAAGCCACCTGGACAAGA AAA -3’. chemokine (C-X-C motif) ligand 2 (CXCL2) (Accession: NM_001001861.1): Forward 5’-CGGAAGTCATAGCCACTCTCAA-3’, Reverse 5’-CAGTAGCCAGTAAGTTTCCTCCATC-3’. Vascular cell adhesion molecule 1(VCAM-1) (Accession: NM_213891.1): Forward 5’- AGCACTTTCAGGGAGGACACA -3’, Reverse 5’-AACGGCAAACACCATCCAA-3’. GAPDH (Accession: NM_001206359.1): Forward 5’- TCCCTGCTTCTACCGGCGCT -3’, Reverse 5’- ACACGTTGGGGGTGGGGACA -3’.

PCR was performed using the following cycling conditions: 95 °C for 30 s, 40 cycles of denaturation at 95 °C for 15 s, and extension at 60 °C for 30 s. All samples were run in triplicate. The relative abundance of target mRNA was determined with the comparative cycle threshold method.

### Western blot analysis

The frozen kidney tissues were lysed with a RIPA lysis buffer (50 mM Tris-Cl [pH 7.6], 150 mM NaCl, 1% NP-40, 0.1% SDS, 0.5% deoxycholic acid, 1 μg/mL leupeptin, 1 μg/mL aprotinin, and 0.5 mM phenylmethylsulfonyl fluoride) and were centrifuged at 12,000 g at 4 °C for 30 min to obtain the cellular proteins in the supernatant. Equal amounts of proteins from each sample were resolved by SDS-PAGE, transferred to NC membranes, blocked with 5% skim milk for 1 h at room temperature, and probed with the following primary antibodies at 4 °C overnight: rabbit anti-LC3 polyclonal antibody (Sigma, L7543) at 1:2000, mouse anti-actin monoclonal antibody (Sigma, A4700) at 1:5000, rabbit anti-pink1 polyclonal antibody (Santa Cruz Biotechnology, sc-33796) at 1:200, rabbit anti-parkin polyclonal antibody (Santa Cruz Biotechnology, sc-30130) at 1:1000, mouse anti-ubiquitin monoclonal antibody (MABtech, MAB1510) at 1:1000, and mouse anti-p62 monoclonal antibody (Santa Cruz Biotechnology, sc-55603) at 1:200, rabbit anti-Bnip3 polyclonal antibody (Abcam, ab38621) at 1:1000, rabbit anti- HIF1α polyclonal antibody (Santa Cruz Biotechnology, sc-10790) at 1:500, rabbit anti-AMBRA1 polyclonal antibody (Abcam, ab72098) at 1:250, mouse-anti-γ-H2AX monoclonal antibody (Abcam, ab11174) at 1:500, mouse-anti-4HNE monoclonal antibody (4-hydroxy-2-nonenal, Abcam, ab46546) at 1:500 and rabbit anti-carbonylated proteins polyclonal antibody(Cell Biolabs, STA-308) . Blots were subsequently probed with horseradish peroxidase- conjugated anti-mouse or anti-rabbit IgG (Santa Cruz Biotechnology, sc-2096, sc-2963) at 1:1000–5000. Immunoreactive bands were visualized by enhanced chemiluminescence, and densitometry was performed using Quantity One software (Bio-Rad Laboratories).

### Immunohistochemistry

The kidneys were fixed in 10% formaldehyde overnight at 4 °C and processed for paraffin-embedding following standard procedures. Sections were cut at 3-μm thicknesses. For immunohistochemical analysis, some tissue sections were subjected to antigen retrieval by microwaving for 10 or 15 min in 10 mM sodium citrate buffer [pH 6.0]. Endogenous peroxidase activity was blocked by incubation with 3% hydrogen peroxide for 10 min. After PBS washing, sections were incubated with 1.5% normal goat serum for 20 min, followed by incubation with mouse monoclonal anti-8-OHdG antibody (1:50; Santa Cruz Biotechnology Inc., sc-66036) overnight at 4 °C. After three washes with PBS, the samples were incubated with biotin-conjugated goat anti-mouse IgG for 30 min at room temperature. After washing in PBS, the sections were incubated with streptavidin-conjugated peroxidase 30 min at room temperature. After PBS washing, the sections were incubated with DAB followed by examination under the microscope.

### Transmission electron microscopy

Kidneys were cut into tissue blocks (1 mm^3^) and fixed in 2.5% glutaraldehyde in 0.01 mol/L phosphate buffer at 4 °C, followed by 2% osmium tetroxide. They were then dehydrated in a series of graded ethanol solutions. Ethanol was then substituted with propylene oxide and the tissue was embedded in epoxy resin. Ultrathin sections were double-stained with uranyl acetate and lead and examined under a JEM1200EX transmission electron microscope (JOEL) at 80 kV.

### Statistical analyses

Analyses of all data were performed using the SPSS ver. 13.0 (SPSS, Chicago, IL, USA) software. Data are expressed as means ± standard deviation (SD). Comparisons among groups were made using analysis of variance. Values of P < 0.05 were considered to indicate statistical significance.

## Results

### Gentamicin causes acute kidney injury in minipigs

#### Dose-response studies

**Figure 1:**
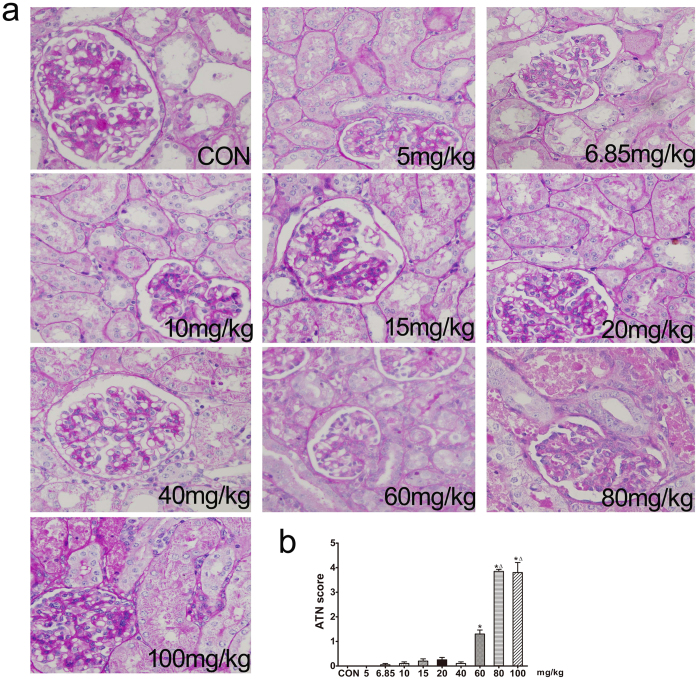
Effects of various doses of gentamicin on renal histology in minipigs. (**a**) Representative photographs of kidney sections from animals given various doses of gentamicin. Periodic acid-Schiff staining. 400×. CON: control group. N = 3 animals per group. (**b**) Renal damage was semiquantitatively scored as described in the Methods section. ATN: acute tubular necrosis. *p < 0.05 *vs*. CON, Δ*p* < 0.05 *vs.* 60 mg/kg gentamicin.

In a gentamicin dose-response study, the blood urea nitrogen (BUN) and serum creatinine levels of minipigs were significantly higher than control values only at drug doses of 80 and 100 mg/kg (Table 1), which were sufficient to produce AKI. Gentamicin-induced renal lesions included epithelial cell necrosis, degeneration, and regeneration of the proximal convoluted tubules. Dilatation of the tubules, protein casts in the tubular lumina, glomerular vacuolization, and interstitial lymphocytic infiltration, were also observed (Fig. 1). The renal histopathological scores indicated that the extent of renal injury was significant, compared to controls, after treatment with gentamicin (60, 80, and 100 mg/kg).

#### Time course studies

**Figure 2:**
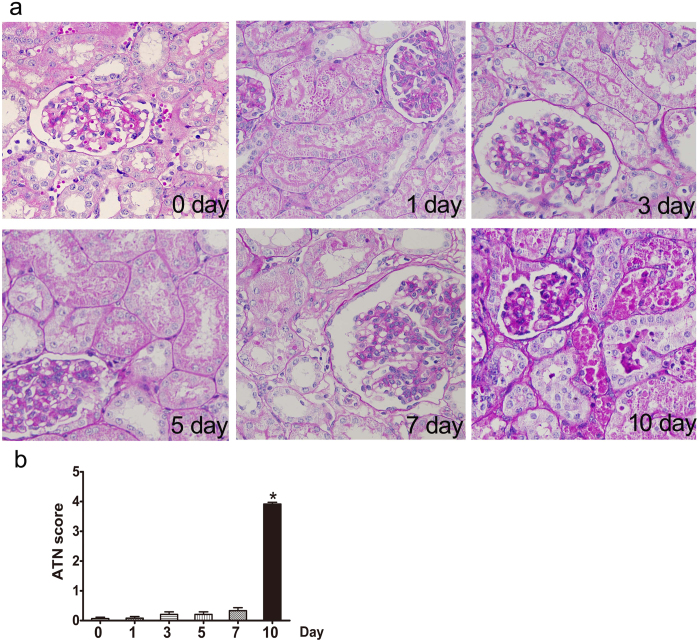
Effects of various intervals of gentamicin on renal histology in minipigs. (**a**) Representative photographs of kidney sections from animals treated with various intervals of gentamicin. Periodic acid-Schiff staining. 400×. CON: control group. N = 6 animals per group. (**b**) Renal damage was semiquantitatively scored as described in the Methods section. ATN: acute tubular necrosis. * *p*< 0.05 *vs.* day 0.

In a gentamicin time-course study, BUN and serum creatinine levels were significantly higher than control values only in the 10-day group (Table 2). Simultaneously, epithelial cell necrosis, degeneration, and regeneration of the proximal convoluted tubules were observed, and renal histopathological scoring indicated significant renal injury in that group, compared to controls (Fig. 2).

### The role of autophagy in gentamicin-induced nephrotoxicity

#### Gentamicin inhibits autophagic degradation in minipig kidneys

**Figure 3:**
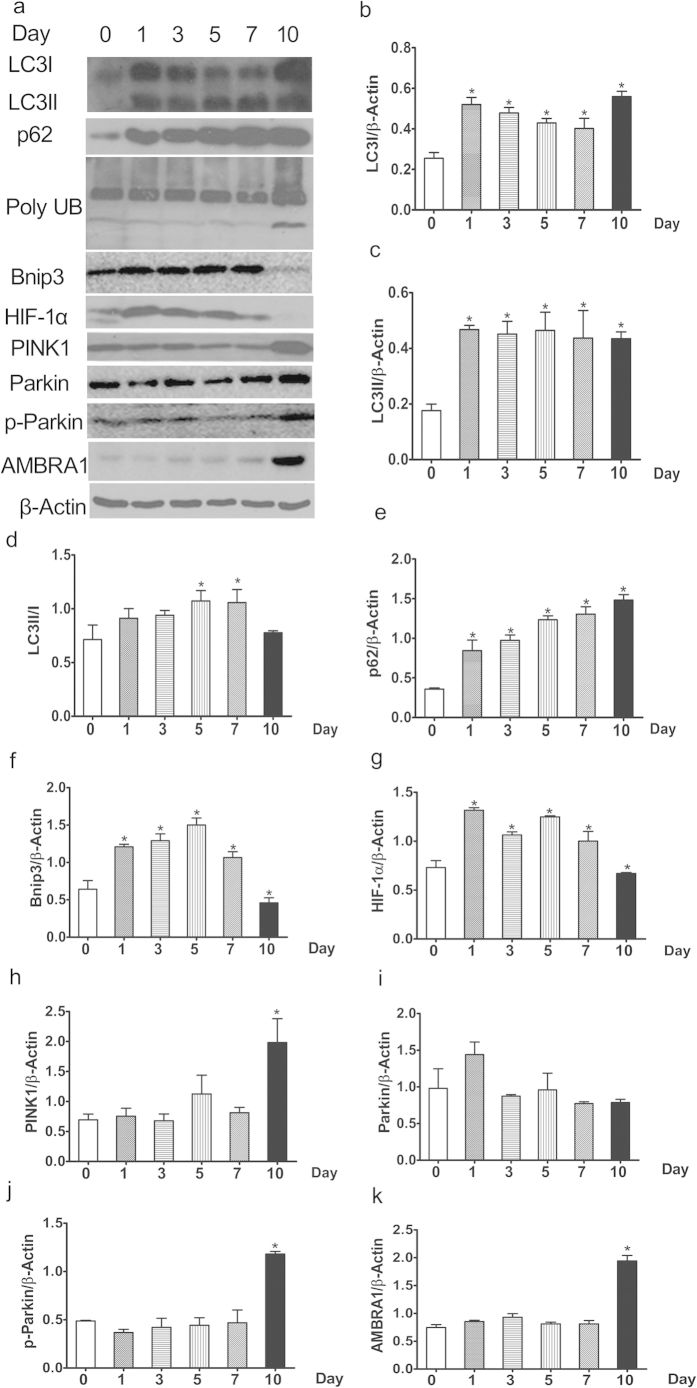
Effects of various intervals of gentamicin on autophagy in minipig kidneys. (**a**) The expression levels of LC3, p62/SQSTM1, poly UB, Bnip3, HIF-1α, PINK1, Parkin, p-Parkin and AMBRA1 in kidney extracts of animals treated with various intervals of gentamicin were quantified by Western blotting. 0, 1, 3, 5, 7 and 10 represent the day 0, 1, 3, 5, 7 and 10 groups, respectively. The gels have been run under the same experimental conditions. (**b–k**) Quantitative band density measurements for LC3, p62/SQSTM1, poly UB, Bnip3, HIF-1α, PINK1, Parkin, p-Parkin and AMBRA1. All data are presented as means ± SDs (n = 6). *p < 0.05 *vs.* day 0.

Autophagic flux refers to all autophagic processes including the formation of autophagosomes, the fusion of autophagosomes with lysosomes, and the subsequent breakdown thereof. P62/SQSTM1 is a ubiquitin-binding protein that interacts with LC3 to mediate the degradation of polyubiquitinated protein aggregates and mitochondria, via the autophagy-lysosome pathway, in mammalian cells. Cellular survival is thus enhanced, because p62/SQSTM1 is sequestered in autophagosomes upon direct interaction with LC3^20^. Therefore, p62/SQSTM1 and polyubiquitinated protein aggregates (poly UB) may act as markers reflecting autophagic degradation activity. Cellular increases in the levels of p62/SQSTM1 and poly UB reflect a decrease in autophagic degradation activity. We found that the expression levels of p62/SQSTM1 and poly UB were significantly increased in 1-, 3-, 5-, 7-, and 10-day kidneys compared to day-0 kidneys (Fig. 3), indicating that gentamicin inhibits autophagic degradation in the kidney.

LC3 is the mammalian homolog of yeast Atg8 and is essential for autophagosomal formation. Precursor LC3 can be digested by Atg4 to form LC3-I, which is then activated by Atg7 to form membrane-bound LC3-II. The latter protein is an autophagosomal membrane marker, and LC3-II/I levels are associated with autophagic flow. We first measured the expression levels of LC3 via Western blotting. Expression of both LC3-I and LC3-II was significantly increased in 1-, 3-, 5-, 7-, and 10-day compared to day 0 kidneys (Fig. 3).

### Gentamicin inhibits mitochondrial autophagic activity in minipig kidneys

Regulation of autophagy by hypoxia-inducible factor 1α (HIF-1α) and a transcriptional target thereof, the “BCL-2 19 kDa interacting protein 3” (Bnip3), was first shown in embryonic fibroblasts of HIF-1α knockout mouse cells subjected to hypoxia^21^. The Bcl2/adenovirus E1B 19-kDa interacting protein 3 (Bnip3) is an atypical “BH3-only” protein linked to mitochondrial dysfunction and cell death. Bnip3 is also a potent inducer of mitochondrial autophagy, which is a protective response^22^. Bnip3 is also induced in an HIF-1α-dependent manner. This HIF-1α-Bnip3 association may contribute to regulation of autophagy in renal tubular cells^23^. Expression of both Bnip3 and HIF-1α was significantly increased in 1-, 3-, 5-, and 7-day kidneys, but clearly decreased in 10-day kidneys, compared to day-0 kidneys (Fig. 3).

PINK1 spans the outer mitochondrial membrane, with the kinase domain facing into the cytoplasm, and is rapidly degraded in healthy mitochondria. PINK1 interacts directly with Parkin in a manner that does not depend on the activity of either PINK1 kinase or Parkin E3-ligase^24^,^25^. The cytoplasm-facing PINK1 kinase domain can phosphorylate Parkin, causing the latter protein to be translocated to mitochondria. Also, phosphorylated Parkin (p-Parkin) ubiquitinates mitochondrial substrates. PINK1 is a sensor of damaged mitochondria. We found that the expression of PINK1 was significantly increased only in 10-day kidneys (compared with day 0 kidneys; Fig. 3).

Parkin is considered to be a substrate of the mitochondrial E3 esterase and may induce mitochondrial autophagy. One such pathway appears to be activated by Parkin after translocation thereof from the cytosol to (specifically) dysfunctional mitochondria^23^. Parkin is involved in removal of such mitochondria. We found that although the Parkin levels were similar in 0-, 1-, 3-, 5-, 7-, and 10-day kidneys, the p-Parkin levels were significantly higher only in 10-day than day-0 kidneys (Fig. 3).

Autophagy-promoting protein AMBRA1 (“activating molecule in Beclin1-regulated autophagy”) binds to depolarized mitochondria in a Parkin-dependent manner. Overexpression of AMBRA1 enhances removal of depolarized mitochondria, but only in the presence of Parkin^26^. We found that expression of AMBRA1 was significantly increased only in 10-day compared to day 0 kidneys (Fig. 3).

### Gentamicin induces oxidative damage in mini-pig kidneys

**Figure 4:**
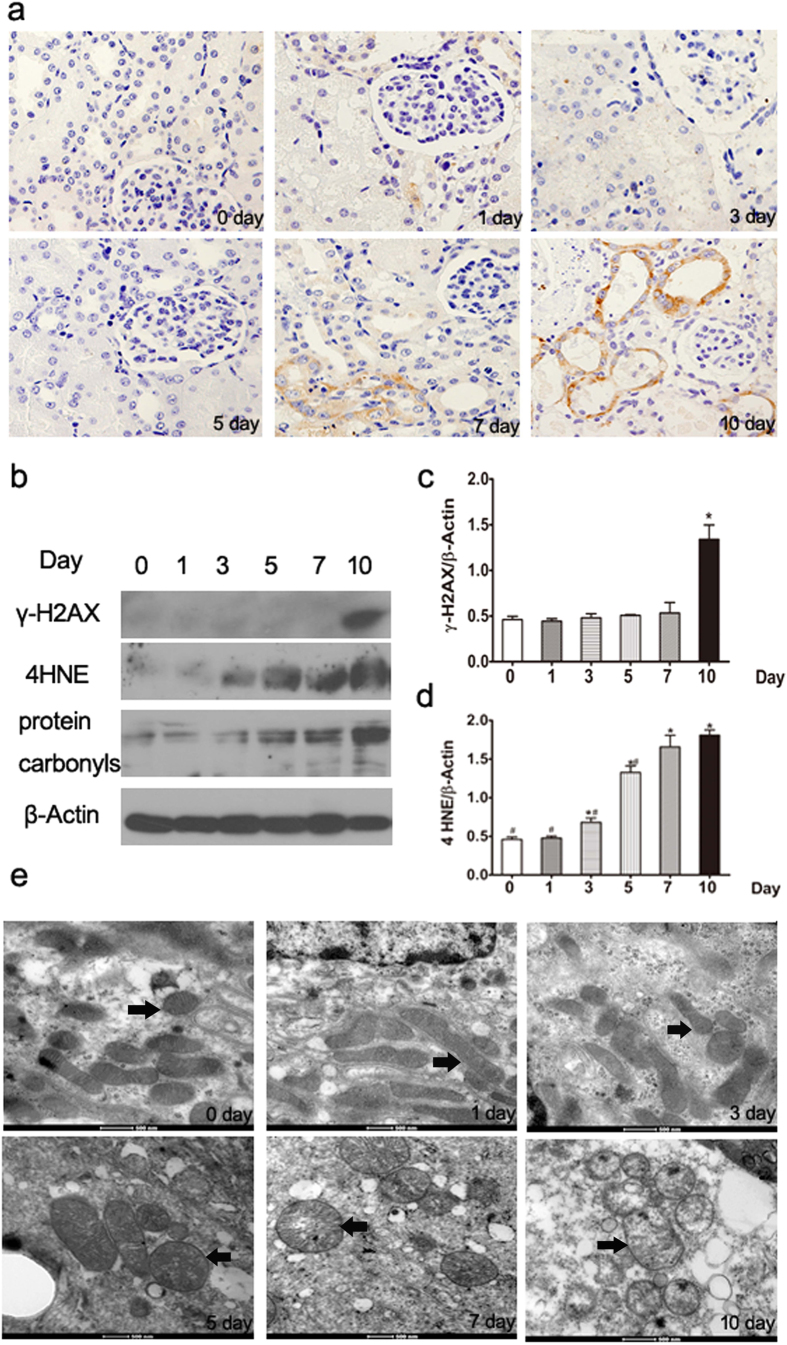
Effects of various intervals of gentamicin on the extent of oxidative damage in minipig kidneys. (**a**) Immunohistochemical staining for 8-OHdG in the kidneys of gentamicin-treated minipigs (400×). (**b**) The levels of γ-H2AX, 4HNE, and protein carbonyls in kidney extracts of gentamicin-treated pigs were measured by Western blotting. 0, 1, 3, 5, 7 and 10 represent the day 0, 1, 3, 5, 7 and 10 groups, respectively. The gels have been run under the same experimental conditions. (**c,d**) Quantitative band density analysis of γ-H2AX and 4HNE. Protein expression data are presented as means ± SDs (n = 6). *p < 0.05 *vs.* day 0. (**e**) Transmission electron microscopy (TM) of mitochondrial structures in renal tissues of gentamicin-treated pigs. Black arrows indicate the mitochondria.

Oxidative stress caused by reactive oxygen species (ROS) can damage both macromolecules including DNA, proteins, and lipids; and mitochondrial structure. To characterize the DNA damage induced by gentamicin in mammalian cells, we used Western blotting to quantify the levels of γ-H2AX, a core histone protein that becomes phosphorylated when DNA is damaged. The level thereof was consistently and significantly increased in 10-day kidneys compared with day-0 kidneys (Fig. 4). Also, we quantified the levels of 8-hydroxy-2′-deoxyguanosine (8-OHdG), an oxidized byproduct of DNA. As was true of γ-H2AX, the levels of 8-OHdG were significantly elevated after 10 days of gentamicin treatment (Fig. 4).

To further explore the nature of cellular oxidative damage, we measured the levels of carbonylated proteins and 4-hydroxy-2-nonenal (4-HNE), a product of lipid peroxidation caused by oxidative damage. We found that the levels of these materials were the highest in 10-day kidneys, compared with kidneys obtained at other times (Fig. 4).

### Gentamicin causes mitochondrial oxidative damage in minipig kidneys

We further explored the effects of gentamicin on mitochondrial structure via transmission electron microscopy. Obvious injury was apparent, including swelling and disintegration of cristae, in 10-day kidneys (Fig. 4).

### Gentamicin promotes renal cell apoptosis in minipig kidneys

**Figure 5:**
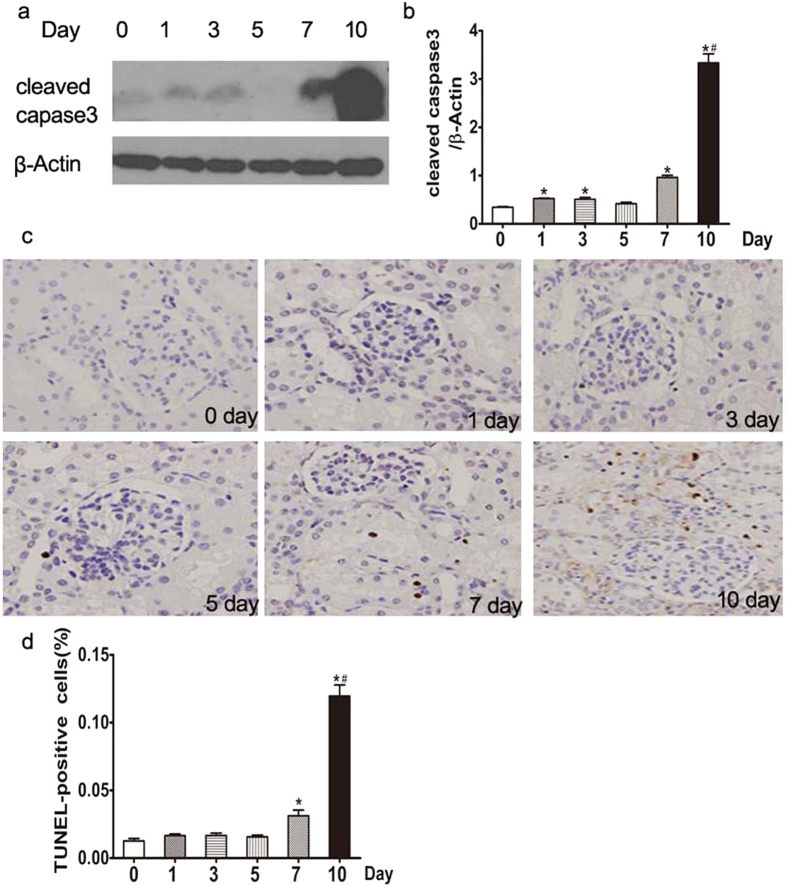
Effects of various intervals of gentamicin on apoptosis in minipig kidneys. (**a**) The expression levels of cleaved caspase-3 in kidney extracts of gentamicin-treated pigs were measured by Western blotting. 0, 1, 3, 5, 7 and 10 represent the day 0, 1, 3, 5, 7 and 10 groups, respectively. The gels have been run under the same experimental conditions. (**b**) Quantitative band density analysis of cleaved caspase-3 levels. Protein expression data are presented as means ± SDs (n = 6). *p < 0.05 *vs.* day 0. (**c**) Representative photographs of kidney cortical specimens, subjected to the TUNEL assay, from pigs treated with gentamicin (400×). (**d**) The numbers of TUNEL (+) cells were counted as described in the Methods section. *p < 0.05 *vs.* day 0. # *p* < 0.05 *vs.* day 7.

The levels of cleaved caspase-3 were significantly increased in 1-, 3-, 7-, and 10-day kidneys compared with day-0 kidneys. The numbers of terminal uridine nick-end-labeled (TUNEL)-positive cells were significantly higher in 7- and 10-day kidneys compared with day-0 kidneys, being greatest in 10-day kidneys (Fig. 5).

### Gentamicin increases the levels of proinflammatory cytokines in minipig kidneys

**Figure 6:**
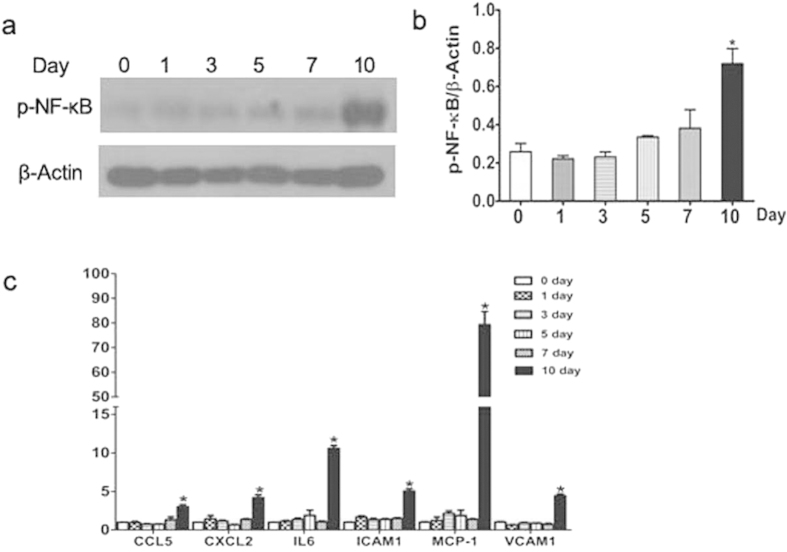
Expression levels of p-NF-κB, ICAM-1, CXCL2, IL-6, VCAM-1, MCP-1, and CCL-5 in gentamicin-treated minipig kidneys. (**a**) The levels of p-p65 (NF-κB) in kidney extracts of gentamicin-treated pigs were measured by Western blotting. 0, 1, 3, 5, 7 and 10 represent the day 0, 1, 3, 5, 7 and 10 groups, respectively. The gels have been run under the same experimental conditions. (**b**) Quantitative analysis of p-p65 (NF-κB) band densities. Protein expression data are presented as means ± Ds (n = 6). *p < 0.05 *vs.* day 0. (**c**) The levels of mRNAs encoding ICAM-1, CXCL2, IL-6, VCAM-1, MCP-1, and CCL-5 mRNA in the kidneys of gentamicin-treated minipigs were measured by quantitative PCR. Data are presented as means ± SDs (n = 6). *p < 0.05 *vs.* day 0.

Compared to the day-0 group, the expression levels of nuclear factor (NF)-κB (p65); and inflammatory cytokines including monocyte chemoattractant protein-1/chemokine (C-C motif) ligand 2 (MCP-1/CCL2), macrophage inflammatory protein-2/C-X-C motif chemokine 2 (MIP-2/CXCL2), CCL-5, vascular cell adhesion molecule (VCAM)-1, interleukin (IL)-6, and intercellular adhesion molecule (ICAM)-1/cluster of differentiation (CD) 54, were significantly increased in 10-day kidneys (Fig. 6).

### Activation of autophagy by rapamycin reduces gentamicin-induced AKI in minipig kidneys

#### Effect of rapamycin on renal function

**Figure 7:**
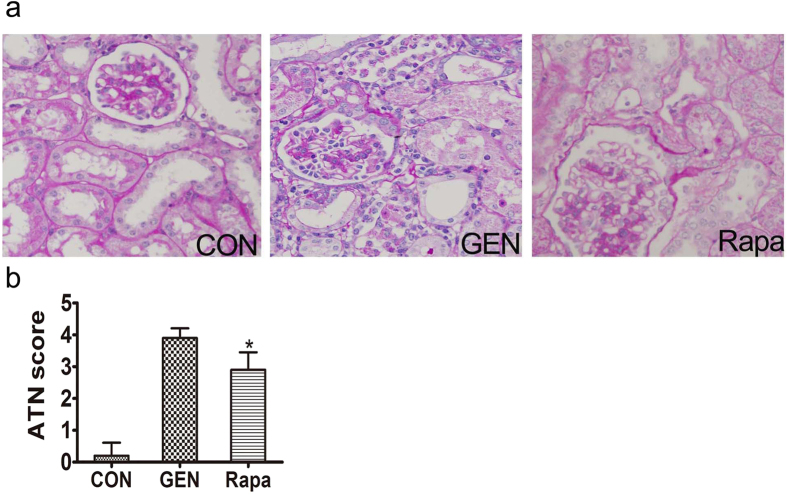
Effect of rapamycin on the renal histology of gentamicin-treated AKI minipigs. (**a**) Representative photographs of kidney sections from animals treated with gentamicin or/and rapamycin. Periodic acid-Schiff staining. 400×. CON: control group. n = 6 animals per group. (**b**) Renal damage was semiquantitatively assessed as described in the Methods section. ATN: acute tubular necrosis. *p < 0.05, *vs*. GEN.

In the rapamycin group, the levels of BUN and serum creatinine were significantly lower than those in 10-day kidneys (Table 3). Rapamycin significantly inhibited gentamicin-induced tubular necrosis and cast formation. The histological scores of kidney sections from gentamicin-treated animals which also received rapamycin were significantly lower than those of animals receiving gentamicin alone (Fig. 7).

#### Effect of rapamycin on autophagy in the kidneys of gentamicin-treated minipigs

**Figure 8:**
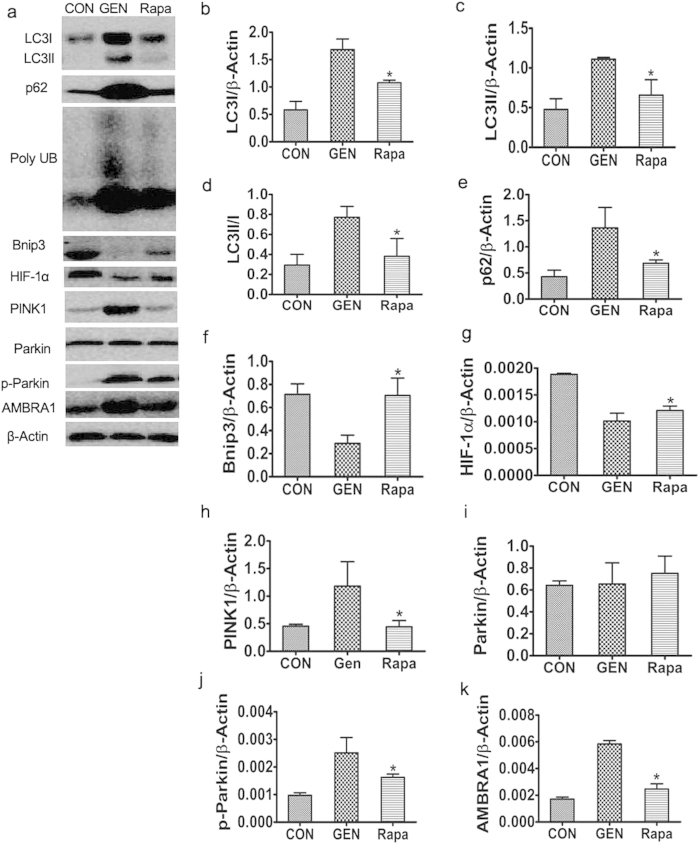
Effect of rapamycin on autophagy in gentamicin-treated AKI minipigs. (**a**) The levels of LC3, p62/SQSTM1, poly UB, Bnip3, HIF-1α, PINK1, Parkin, p-Parkin and AMBRA1 in kidney extracts of animals treated with various doses of gentamicin were quantified by Western blotting. CON: the control group, GEN: the gentamicin-treated group, Rapa: the gentamicin and rapamycin treated group. The gels have been run under the same experimental conditions. (**b–k**) Quantitative measurements of band densities of LC3, p62/SQSTM1, poly UB, Bnip3, HIF-1α, PINK1, Parkin, p-Parkin and AMBRA1. Protein expression data are presented as means ± SDs (n = 6). *p < 0.05 *vs.* GEN.

The levels of LC3-I, LC3-II, p62, Poly UB, PINK1, p-Parkin, and AMBRA1 were significantly lower in the kidneys of rapamycin- than gentamicin-treated animals. The Parkin levels were, however, similar. Bnip3 and HIF-1α levels in the kidneys of rapamycin were higher than gentamicin-treated animals (Fig. 8).

#### Effect of rapamycin on gentamicin-induced oxidative damage to minipig kidneys

**Figure 9:**
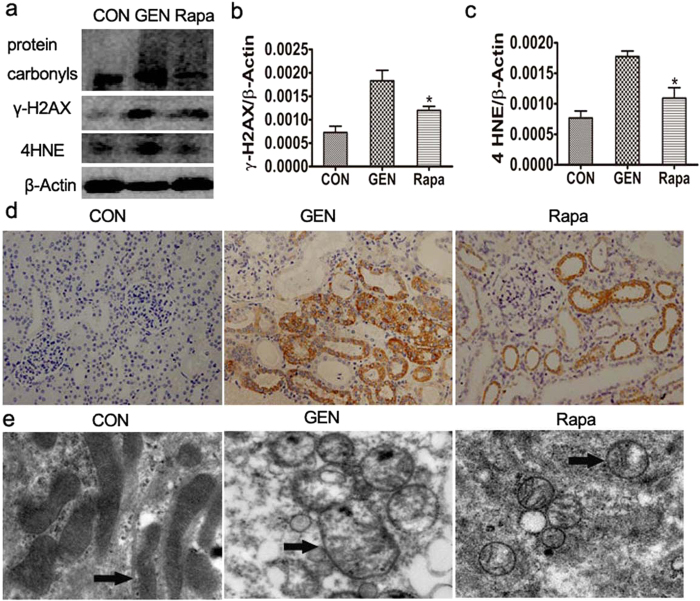
Effect of rapamycin on oxidative damage in gentamicin-treated AKI minipigs. (**a**) The levels of γ-H2AX, 4HNE, and protein carbonyls in kidney extracts from gentamicin-treated pigs were measured by Western blotting. CON: the control group, GEN: the gentamicin-treated group, Rapa: the gentamicin and rapamycin treated group. The gels have been run under the same experimental conditions. (**b,c**) Quantitative analysis of the band densities of γ-H2AX and 4HNE. Protein expression data are presented as means ± SDs (n = 6). *p < 0.05 *vs.* GEN. (**d**) Immunohistochemical staining of 8-OHdG in the kidneys of gentamicin-treated pigs. 400×. (**e**) Transmission electron microscopy (TM) of mitochondrial structures in the renal tissues of gentamicin- or/and rapamycin-treated pigs. Black arrows indicate the mitochondria.

The levels of 8-OHdG and γ-H2AX were significantly elevated after 10 days of gentamicin treatment (Fig. 9). To further explore the accumulating cellular oxidative damage, we measured the levels of protein carbonyls and 4-HNE, a lipid peroxidation product resulting from oxidative damage. We found that the levels of these materials were clearly lower in the kidneys of rapamycin- than gentamicin-treated kidneys (Fig. 9). Thus, rapamycin inhibited the mitochondrial structural damage, including swelling and disintegration of cristae, caused by gentamicin.

#### Effect of rapamycin on gentamicin-induced apoptosis and inflammation in minipig kidneys

**Figure 10:**
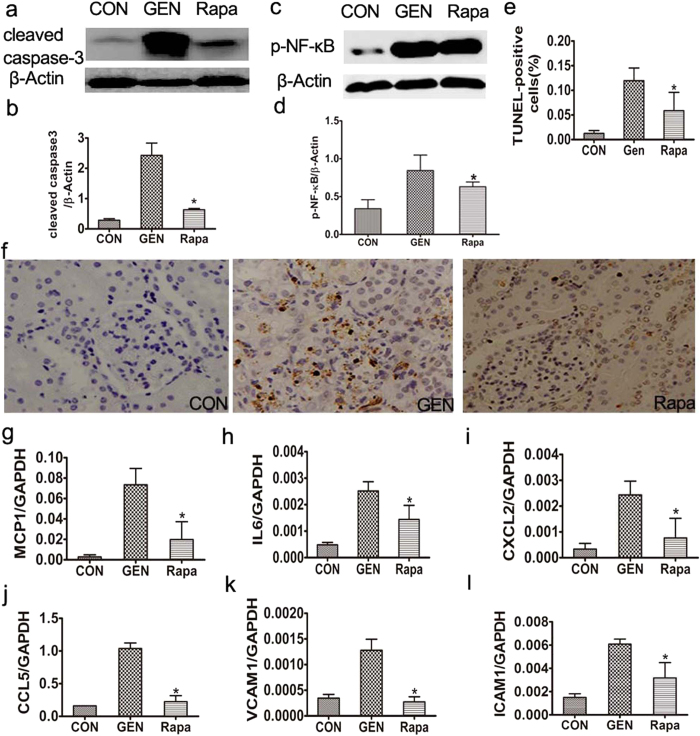
Effects of rapamycin on apoptosis and inflammation in gentamicin-treated AKI minipigs. (**a**) The levels of cleaved caspase-3 in kidney extracts of gentamicin-treated pigs were measured by Western blotting. CON: the control group, GEN: the gentamicin-treated group, Rapa: the gentamicin and rapamycin treated group. The gels have been run under the same experimental conditions. (**b**) Quantitative analysis of the band densities of cleaved caspase-3. Protein expression data are presented as means ± SDs (n = 6). *p < 0.05 *vs.* GEN. **(c)** The levels of p-NF-κB in kidney extracts of gentamicin-treated pigs were measured by Western blotting. CON: the control group, GEN: the gentamicin-treated group, Rapa: the gentamicin and rapamycin treated group. **(d)** Quantitative analysis of the band densities of p-NF-κB. Protein expression data are presented as means ± SDs (n = 6). *p < 0.05 *vs.* GEN. (**e**) Representative photographs of kidney cortical sections, on which the TUNEL assay had been performed, from pigs treated with gentamicin. 400×. (**f**) The numbers of TUNEL (+) cells were scored as described in the Methods section. *p < 0.05 *vs.* GEN. # p < 0.05 *vs.* day 7. (**g–l**) The expression levels of mRNAs encoding MCP-1, IL-6, CXCL2, CCL-5, VCAM-1, and ICAM-1 in minipig kidneys were measured by quantitative PCR. Data are presented as means ± SDs (n = 6). *p < 0.05 *vs.* GEN.

The numbers of terminal uridine nick end-labeled (TUNEL)-positive cells, and the levels of cleaved caspase-3, were significantly lower in the kidneys of rapamycin- than gentamicin-treated animals (Fig. 10). Compared to the gentamicin-treated animals, the expression level of nuclear factor (NF)-κB (p65) was significantly decreased in the rapamycin -treated animals (Fig. 10).

Compared to the latter minipigs, the levels of inflammatory cytokines including monocyte chemoattractant protein-1/chemokine (C-C motif) ligand 2 (MCP-1/CCL2), macrophage inflammatory protein-2/C-X-C motif chemokine 2 (MIP-2/CXCL2), CCL-5, vascular cell adhesion molecule (VCAM)-1, interleukin (IL)-6, and intercellular adhesion molecule (ICAM)-1/cluster of differentiation (CD) 54, were significantly lower in the kidneys of rapamycin-treated animals (Fig. 10).

## Discussion

The kidney has both filtration and secretion functions, making it the main excretory organ of many drugs. Gentamicin is widely used to treat infectious diseases caused by gram-negative bacteria in animals and humans. However, nephrotoxicity has limited its application. Therefore, an in-depth understanding of gentamicin-induced kidney damage mechanisms is essential. Nephrotoxicity models have been established in mouse and rat, but due to genetic, biochemical and physiological differences between rodents and humans, it is necessary to establish an aminoglycoside kidney damage model using large animals, such as pigs or monkeys, which are more similar to human beings.

Oxidative stress plays a critical role in the pathophysiology of bactericidal antibiotic-induced injury. Bactericidal antibiotics include quinolones, aminoglycosides, and β-lactams^27^. Bactericidal antibiotics (quinolones, aminoglycosides, and β-lactams) caused mitochondrial dysfunction and ROS overproduction in mammalian cells, ultimately leading to the accumulation of oxidative tissue damage. Autophagy, a lysosomal degradation pathway, plays a crucial role in removing protein aggregates as well as damaged organelles to maintain intracellular homeostasis in renal pathophysiology^28^,^29^. Autophagy is particularly important for maintenance of post-mitotic cells, such as podocytes, which have only a limited capacity for regeneration^29^.

The exact mechanism of nephrotoxicity caused by aminoglycoside antibiotics, such as gentamicin, is not yet clear. In this study, we investigated the role of autophagy, particularly mitochondrial autophagy, in the pathogenesis of gentamicin nephrotoxicity. First, we successfully established a gentamicin-induced nephrotoxicity model in mini-pigs, and found that serum creatinine and blood urea nitrogen were significantly increased in the 80 and 100 mg/kg gentamicin-injected groups. Renal pathological analysis revealed significant renal tubular epithelial cell necrosis in the 80 and 100 mg/kg gentamicin-injected groups. Although the ATN score in the 60 mg/kg gentamicin-injected group was significantly increased compared with the CON group, serum creatinine and blood urea nitrogen were not significantly increased. Additionally, all of the mini-pigs were dead on the third day after 100 mg/kg gentamicin treatment (i.m.). Therefore, in the gentamicin-induced injury study time course, we decided to observe changes in renal function, autophagy, inflammation, apoptosis, and oxidative damage at 0, 1, 3, 5, 7 and 10 days after 80 mg/kg gentamicin treatment (i.m.).

We found a compensatory increase in autophagosome formation-related proteins (LC3I and LC3II) in mini-pigs administered gentamicin; however, the increased expression of p62/SQSTM1 and polyubiquitin aggregates indicated that autophagic degradation capacity was decreased in the kidneys of gentamicin-treated mini-pigs. Bnip3 was identified as a potent inducer of autophagy in cells. Bnip3 is an important regulator of mitochondrial turnover via autophagy in the myocardium^30^. HIF-1α-Bnip3 may contribute to the regulation of autophagy in renal tubular cells^31^. In our study, we observed that Bnip3 and HIF-1α were significantly elevated in the early stage of gentamicin treatment, and markedly decreased in the 10-day group. These results indicated that mitophagy was activated in the early stage, while mitophagy function declined in the gentamicin-induced AKI. Animal studies showed that ER stress or oxidative stress induces adaptive autophagy upregulation in the early phase, which helps to restore intracellular homeostasis by disposing of a number of harmful molecules, such as unfolded or misfolded proteins in the ER lumen, cytosolic proteins damaged by ROS, or even dysfunctional ER and mitochondria^32^,^33^,^34^. Upon mitochondrial damage, mitochondria-localized PINK1 recruits and phosphorylates the E3-ligase, parkin, to mitochondria. At the same time, parkin can polyubiquitinate voltage-dependent anion channel (VDAC)1, followed by p62 binding to VDAC1 on the mitochondria and ATG8/LC3/GABARAP on the developing autophagosome, resulting in mitochondrial sequestration and removal by the autophagic machinery^26^. Parkin reportedly surveys mitochondrial quality by translocating to depolarized mitochondria and inducing their selective macroautophagic removal (mitophagy). In addition, p-parkin interacts with AMBRA1, a protein that promotes autophagy in the vertebrate central nervous system^35^. Prolonged mitochondrial depolarization strongly increases the interaction of parkin with AMBRA1. We found that mitophagy markers, PINK1, p-parkin and AMBRA1, were markedly increased in 10-day gentamicin-treated mini-pig kidneys, indicating that mitochondrial damage can significantly enhance mitophagy activity in the late stage of gentamicin-induced AKI in mini-pig kidney.

Mitophagy is the only pathway that removes damaged mitochondria from cells. Therefore, inhibition of mitochondrial autophagy function leads to the accumulation of damaged mitochondria. Because mitochondria are the main intracellular site for ROS production, damaged mitochondria accumulation will lead to more ROS production, and further promote pro-inflammatory cytokine expression and apoptosis.

In humans, it is likely that oxidative stress and related oxidative cellular damage induced by bactericidal antibiotics underlie many of the adverse side effects associated with these antibiotics^36^,^37^,^38^. In our experiment, oxidative damage markers, including 4HNE, protein carbonyls, γ-H2AX and 8-OHdG, were significantly elevated in the 10-day group. Meanwhile, the results showed obvious injury to mitochondrial structures, such as swelling and disintegration of cristae in the 10-day gentamicin-treated group. Under normal circumstances, the damaged mitochondria are degraded by autophagy. If the impaired mitochondria are not removed, they can generate more ROS, which further aggravates oxidative damage, forming a vicious cycle^39^,^40^. Therefore, mitochondria play a central role in tissue damage caused by gentamicin^40^.

It was shown that gentamicin can induce apoptosis and inflammation in renal tubular cells^41^,^42^,^43^,^44^. In accordance with previous reports, our results showed that the level of cleaved caspase-3 and the number of TUNEL(+) cells were significantly increased in gentamicin-induced AKI. Meanwhile, the expression levels of nuclear factor (NF)-κB (p65) and inflammatory cytokines were significantly elevated in the gentamicin-induced AKI mini-pigs.

Characterization of bactericidal antibiotic effects on mammalian cells points to widespread cellular oxidative damage^27^. According to the results of our experiments, autophagy plays a central role in removing cellular oxidative damage caused by gentamicin. We next identified whether the activation of autophagy by rapamycin protects against gentamicin-induced AKI. Therefore, we explored the possibility that using rapamycin, a pharmacological inhibitor of mTOR, could enhance autophagy to alleviate these deleterious effects. Identifying bactericidal antibiotics as a cause of ROS overproduction and autophagy in mammalian cells provides a basis for developing therapeutic strategies that could help alleviate adverse side effects associated with antibiotics. For instance, by coadministering rapamycin, we showed that autophagy, particularly mitophagy, and renal function could be recovered; oxidative damage, apoptosis and inflammation induced by bactericidal antibiotics could be abrogated. When appropriate for patient health, coadministering a pharmacological autophagy activator could be a simple treatment strategy aimed at preventing cellular oxidative damage. It will be important to extend our investigation to human subjects to confirm our findings, demonstrate their relevance to humans, and maximize the translational value of our work. Epidemiologic studies could provide valuable insight into the clinical implications of antibiotic-induced oxidative damage and define some of the risks associated with bactericidal antibiotic exposure. In humans, it is likely that oxidative stress and related oxidative cellular damage induced by bactericidal antibiotics underlie many of the adverse side effects associated with these antibiotics^36^,^37^,^38^. It will be intriguing to explore these possibilities with appropriate clinical trials, with the goal of developing effective antibacterial therapies with minimal adverse side effects.

## Additional Information

**How to cite this article**: Cui, J. *et al.* Rapamycin protects against gentamicin-induced acute kidney injury via autophagy in mini-pig models. *Sci. Rep.* **5**, 11256; doi: 10.1038/srep11256 (2015).

## Figures and Tables

**Table 1:** **Effect of various doses of gentamicin on renal function in minipigs.**

**(mg/kg)**	**CON**	**5**	**6.85**	**10**	**15**	**20**	**40**	**60**	**80**	**100**
BUN (mmol/L)	3.3 ± 0.8	4.6 ± 0.3	3.9 ± 0.9	5.3 ± 0.5	6.0 ± 1.6	5.2 ± 1.1	7.2 ± 2.7	9.1 ± 1.7	29.4 ± 6.4*	83.9 ± 5.6*#
SCr (μmol/L)	66.0 ± 5.0	90.7 ± 9.6	84.9 ± 2.1	99.4 ± 5.4	108.4 ± 29.1	92.0 ± 8.8	178.7 ± 52.9	156.7 ± 11.9	366.1 ± 78.9*	1137.6 ± 348.3*#

BUN, blood urea nitrogen; Scr, serum creatinine. Values are presented as means ± SD (n = 3). *p < 0.05 *vs*. CON, # *p* < 0.05 *vs.* 80 mg/kg gentamicin.

**Table 2:** **Effect of various intervals of gentamicin on renal function in minipigs.**

**day**	**0**	**1**	**3**	**5**	**7**	**10**
BUN (mmol/L)	3.8 ± 0.9	3.4 ± 0.5	4.4 ± 1.7	4.2 ± 0.6	5.1 ± 1.3	26.7 ± 8.2*
Scr (μmol/L)	80.9 ± 11.9	73.2 ± 15.8	68.2 ± 11.5	69.2 ± 6.8	101.3 ± 55.9	295.2 ± 65.3*

BUN, blood urea nitrogen; Scr, serum creatinine. Values are presented as means ± SD (n = 6). *p *< 0.05, vs.* day 0.

**Table 3:** **Effect of rapamycin on renal function in gentamicin-induced minipig.**

	**CON**	**GEN**	**Rapa**
BUN (mmol/L)	3.8 ± 0.9	26.7 ± 8.2	5.86 ± 2.93*
Scr (μmol/L)	80.9 ± 11.9	295.2 ± 65.3	135.8 ± 33.3*

BUN, blood urea nitrogen; Scr, serum creatinine. CON, control group, GEN, gentamicin, Rapa, rapamycin. Values are presented as means ± SD (n = 6). *p < 0.05, *vs.* GEN.
